# I’ve really struggled but it does not seem to work: adolescents’ experiences of living with ADHD – a thematic analysis

**DOI:** 10.1186/s40359-025-02350-7

**Published:** 2025-01-27

**Authors:** Vendela Zetterqvist, Caisa Öster, Anna Oremark, Lotta Myllys, Jenny Meyer, Mia Ramklint, Johan Isaksson

**Affiliations:** 1 Department of Medical Sciences, Child and Adolescent Psychiatry, Uppsala University, Akademiska sjukhuset, ingång 10, plan, Uppsala, 751 85 Sweden; 2 Center of Neurodevelopmental Disorders, Centre for Psychiatry Research, Department of Women’s and Children’s Health, Karolinska Institute and Stockholm Health Care Services, Stockholm, Sweden

**Keywords:** Attention Deficit/Hyperactivity Disorder, Qualitative research, Interview

## Abstract

**Background:**

In Attention-deficit/hyperactivity disorder (ADHD) the transition from childhood to adolescence encompass changes in symptom manifestation and related challenges. Given the potential negative impact of ADHD on adolescents, and the increased risk for dropping out from treatment, there is a need to understand more about how adolescents experience their condition. The aim of this study was to explore adolescents’ perceptions of how it is to live with ADHD.

**Methods:**

Twenty adolescents (15–17 years old, 12 girls) diagnosed with ADHD who had completed a skills training group were interviewed. Data was analysed using thematic analysis.

**Results:**

Four themes emerged: ‘I find it hard to regulate what I take in and what comes out’, ‘I’ve really struggled but it does not seem to work’, ‘ADHD impacts my vitality and my relationships’, and ‘I can be successful and things are getting better’.

**Conclusion:**

Adolescents with ADHD experience problems with self-regulation with regards to cognitive, emotional and behavioural processes. They describe being either on or off. This difficulty to self-regulate is related to primary and secondary consequences such as stress, anxiety, loss of control, and by extension exhaustion, depressed mood, challenges in social interactions, self-accusations and a tendency of giving up. The experience of ‘being on’ has positive connotations of vigour and creativity. The challenges related to ADHD can to some extent be overcome with time for recovery, coping strategies, and medical treatment. Symptoms of ADHD can also decrease over time.

**Trial registration:**

This qualitative study was not pre-registered.

**Supplementary Information:**

The online version contains supplementary material available at 10.1186/s40359-025-02350-7.

## Introduction

Attention-deficit/hyperactivity disorder (ADHD) is a neurodevelopmental condition that affects 5–7% of children and adolescents around the world, with a two times higher prevalence for boys than girls [1, 2]. The diagnostic criteria for ADHD focus on symptoms of inattentiveness and/or hyperactivity/impulsiveness, resulting in impairment in several domains of life [2]. Adolescents with ADHD are at a significant risk of having lower grades, absenteeism, and school dropout [3, 4], having a harder time developing stable peer relationships [5, 6], and ADHD is related to relational problems, and conflicts with parents and siblings [7, 8]. Moreover, emotional reactivity, difficulties in emotion regulation and co-occurrence of other psychiatric conditions are often present together with the core symptoms of ADHD, adding further to the challenges in adaptive functioning [9, 10]. How the symptoms are expressed and affect the individual often changes as the individual gets older - both as a result of developmental processes, and as a result of changes in demands from the environment/society [11].

Although ADHD is typically diagnosed in childhood or adolescence [12] a large proportion of those affected continue having symptoms and impairment as adults [13]. The condition has predominantly been studied in younger children, using parents or teachers as informants [14], and a growing amount of research covering ADHD in adulthood is emerging [15], while fewer studies have specifically explored the adolescent period. It is known that symptom manifestation and related problems changes from childhood to young adulthood [11, 16]. Symptoms of hyperactivity/impulsivity tend to attenuate or become internalised and more subtle with age, while inattention tends to persist [11]. Although core symptoms decline, difficulties with self-regulation can remain [13], and when compared to children, adolescents with ADHD have worse global functioning, fewer adaptive skills, more emotional problems, somatic complaints, and social problems [17]. In general, adolescents spend a lot of time outside the family and are in a process of separating from their parents [18]. For these reasons, symptoms and related consequences may be less observable for others, and it is therefore relevant to turn to the adolescents themselves for information [19]. Furthermore, adolescents are less likely than school-aged children to utilize evidence-based behavioural and pharmacological treatments for ADHD [20]. Pharmacological treatments are often initiated in childhood by parents [21], but discontinued in adolescence [22]. This suggests that the transition from childhood to adulthood bring important changes in the perceived need for treatment [22], and most likely how the adolescent perceive the condition. A better understanding of the adolescents’ perceived situation is required in order to offer treatments that are in line with their own needs.

Adolescence is a period in life when the individual goes through major changes physically, cognitively, and emotionally. Adolescents develop an increased ability to reason abstractly and systematically plan for the future [23], which enables them to reflect upon themselves and their life situation in a new way, and to form an identity. Identity formation entails the adolescents exploring who they are, who they want to become, what commitments they should stay true to, and where they fit into society [24]. Altogether, it can be argued that the adolescents’ own perspective on ADHD has been given little attention, and qualitative interviews can provide richness to the apprehension of their experiences [25]. However, to date, most studies has been quantitative in nature, focusing on measuring symptom severity and functioning. There is a small body of literature based on interviews capturing the experiences and perceptions of adolescents diagnosed with ADHD, of which many have been published during the last decade (see Ringer [26] for a review). Most of these studies cover perceived consequences of ADHD, including both symptoms (e.g. becoming distracted, daydreaming, lack of behavioural control) and impairments (e.g. academic failures, problems following rules and getting along with others) related to ADHD (e.g. [27–29]). Some studies found that the adolescents also attribute some positive consequences to their condition (e.g. [28, 30]). With regards to identity, the process of receiving the diagnosis ADHD, how the diagnosis is perceived by others, and how it is integrated in the adolescents’ self-perception has been captured in a handful of studies (e.g. [30, 31]). These studies cover stigma and self-stigma [32], but also relief [33], and being given a new understanding [34]. Control and empowerment were identified as themes and discussed in Clancy et al. [35] and in Krueger & Kendall [36]. Strategies for obtaining control were mentioned in several studies, most commonly the use of medication (e.g. [37]), and the support from others (e.g. [38]), but sometimes also self-management strategies (e.g. [39]).

To summarise, there is a need to understand more about how adolescents perceive and manage their disorder, in order to identify the gaps in current mental health services for this age-group, and qualitative research may capture the nuanced perspectives, emotions, and experiences of adolescents with ADHD. The aim of this study was to explore adolescents’ perceptions of how it is to live with ADHD.

## Methods

### Procedure and participants

 Recruitment took place at two child- and adolescent psychiatric outpatient units, among adolescents 15–17 years old diagnosed with ADHD. All participants had recently completed a structured skills training group based on Dialectical Behaviour Therapy, which was adjusted to address challenges related to living with ADHD (for more information about the treatment see Meyer et al. [40]). For the present study recruitment was done in two steps. First the group therapists informed the adolescents about the study during the last session. Later a representative for the research group contacted the adolescents by telephone and asked if they were interested in participating in an interview. The interview was exploratory and covered three topics: [1] how it is to live with ADHD [2], how the adolescents experience and perceive stress and [3] experiences of the group treatment. The findings regarding the second and third topic have already been published elsewhere [41, 42]. Twenty-one adolescents were eligible, of which one declined participation due to lack of time. Twenty participants were thus included, with a mean age of 16.30 years (SD = 0.92), of which 12 were girls, and 8 were boys.

A structured diagnostic procedure based on the Mini International Neuropsychiatric Interview for children and adolescents (MINI Kid; [43]) and the Adult ADHD Self-Report Scale for Adolescents (ASRS-A; [44]) was used to validate the clinical ADHD diagnosis. Clinical psychologists carried out the diagnostic assessment. The ASRS-A is validated scale for measuring ADHD symptoms with scores ranging from 0 to 72, where higher scores indicate more symptoms. See Table 1 for demographic and clinical characteristics of the sample.

**Table 1:** Demographic and clinical characteristics of the adolescents

Characteristic	Descriptive statistics
Females, n	12 (60%)
Age, Mean (SD)	16.30 (0.92)
Clinical diagnosis, n	ADHD-combined, 13 ADHD-inattentive, 5 ADHD (NOS), 2
ASRS-A^a^	
Self-ratings, Mean (SD)	38.65 (15.37)
Parental-ratings, Mean (SD)	36.94 (11.47)
ADHD medication^b^, n	16

*ADHD-combined *Attention-deficit/hyperactivity disorder, combined presentation, *ADHD-inattentive* Attention deficit disorder, predominantly inattentive presentation, *ADHD (NOS)* Attention-deficit/hyperactivity disorder not otherwise specified

^a^Adult ADHD Self-Report Scales for Adolescents

^b^Methylphenidate, atomoxetine, lisdexamfetamine, or guanfacine

The study was conducted in accordance with the Declaration of Helsinki, and the procedure was approved by the Regional Ethical Review Board in Uppsala (2015/257/2). The review board constitutes a state authority. Prior to the interviews, written informed consent was obtained from all participants. According to Swedish legislation 15 is the age limit for consent. In addition, all parents/legal guardians had given written informed consent to the overall study, and an oral informed consent for the interviews specifically. The study planning, data analysis, and results presentation was guided by the consolidated criteria for reporting qualitative studies (COREQ; [45]).

### Interviews

Interviews were conducted by two graduate students with basic clinical education (in psychology and in medicine), who were trained in interviewing technique. The interviewers were not involved in the treatment, and had no prior relationship to the participants. The timing of the interviews was 1–2 weeks posterior to group treatment. The interviews mostly took place at the outpatient units, except one interview, which was conducted via video call, as that participant was unable to attend in person.

The current study targeted the topic “How it is to live with ADHD”. The interview started with the questions *What is it like*,* having ADHD?* and *What is good or bad with ADHD?* The interviewers closely followed the question sequence of the interview schedule (see Supplement material). The narratives were explored in relation to home, school, relationships and wellbeing. Probes and follow-up questions were given when appropriate. The interviews, lasted for 20 to 60 min, were audio recorded and transcribed verbatim. The transcripts were checked against the tapes for accuracy.

### Data analysis – procedure and underlying assumptions

The transcribed interviews were analysed using Thematic analysis, which is a commonly used method of analysis in qualitative research, systemising and describing patterns of meaning (themes) within the data. [46]. Thematic analysis is a flexible method that can be applied to a large variety of research questions, and across a range of theoretical or epistemological approaches [47]. In the current study we applied an inductive/reflexive, experiential, contextualistic meta-theoretic framework. To specify further, the analysis was (a) data-driven, but interpreted by active, subjective researchers, and is therefore considered as reflexive rather than purely inductive, (b) experiential as in making sense of (shared) experiences and meanings ascribed to the phenomena by the participants, and (c) contextualistic as in assuming a knowable world (as opposed to viewing the world as socially or verbally constructed).

The analysis was performed in a stepwise fashion. The transcriptions were read in detail repeatedly, to become familiar with the data and develop a holistic understanding of the adolescents’ experiences. Two independent coders extracted text from the transcriptions in relation to the aim of the study, in an ‘open coding’. The transcription excerpts were condensed into codes (see Table 2 for examples). The two sets of codes were very similar. Differences between the sets were discussed and the sets were merged. All identified codes were first read thoroughly and aggregated into potential themes and sub-themes, in a process involving all authors. The codes and transcriptions were read again to check if the themes were representative for the codes, and the entire data set. This also involved a procedure of reviewing if the entire material was represented in the thematic map. An interpretative process took place where the specifics of each theme were refined, and the overall story of the analysis was created. In this process the relationship between different themes and sub-themes was analysed by going back and forward to the transcribed material. For instance the authors formulated questions to the material like ”Where in the narrative are emotions being described?”

**Table 2:** Example of the relationship between transcription excerpts, codes, sub-themes and themes

Transcription excerpt	Condensed excerpt	Code	Sub-theme	Theme
**… if someone is sitting there fiddling with their hand**,** for example**,** someone might be sitting and typing on their mobile**,** there is no filter**,** I see everything that everyone else does.**	**there is no filter**,** sees everything that everyone does**	**there is no filter**	**Inner chaos**	**I find it hard to regulate what I take in and what comes out**
**… if you read something on the internet or something, it may also be that you have to go back and check what you have actually read.**	**having to go back and check what you have actually read**	**a need to read again**	**Blankness**	
**Just this, not being able to really focus on one and the same thing for long periods of time, or not having interest in anything, and just simply not listening. And then, when you finally find something that you like, that becomes… then you focus on that one hundred per cent**	**not being able to focus and then finally…you are interested and focus one hundred per cent**	**finally you focus fully when interested**	**I am either off or on**	

Several measures were taken to assure the rigour of the analysis. First of all through familiarity with and continual immersion in the data at every step of the analysis. When a categorisation system had been elaborated it was tested for completeness in several ways [25]. All codes were reviewed to see if they could be categorised in the system in a meaningful way. The categorization and construction of themes were considered completed, when the themes were internally consistent and the themes were found to capture the meanings evident in the data as a whole. The authors discussed the themes until consensus was attained.

## Results

In the analysis of the interviews four themes and eleven sub-themes were identified. The four themes were [1] I find it hard to regulate what I take in and what comes out [2] I’ve really struggled but it does not seem to work [3] ADHD impacts my vitality and my relationships [4] I can be successful and things are getting better (see Table 3). All themes and sub-themes are presented and illustrated with quotes from the material (the number of the respondent is indicated with #).

**Table 3:** Thematic ’map’ of the analysis

Theme	Sub-theme
I find it hard to regulate what I take in and what comes out	Inner chaos
	Blankness
	I am either off or on
A lot of effort is required from me	My way of being works against me
	I get drained and exhausted
ADHD impacts my vitality and my relationships	I am tiresome to others, others are tiresome to me
	I resign and blame myself
I can be successful and things are getting better	When I am vigorous and creative
	When I use the inner stress or strong emotions as motivation
	With time for recovery, good strategies and the help of medicine
	Certain symptoms have decreased over time

### I find it hard to regulate what I take in and what comes out

**Fig. 1:**
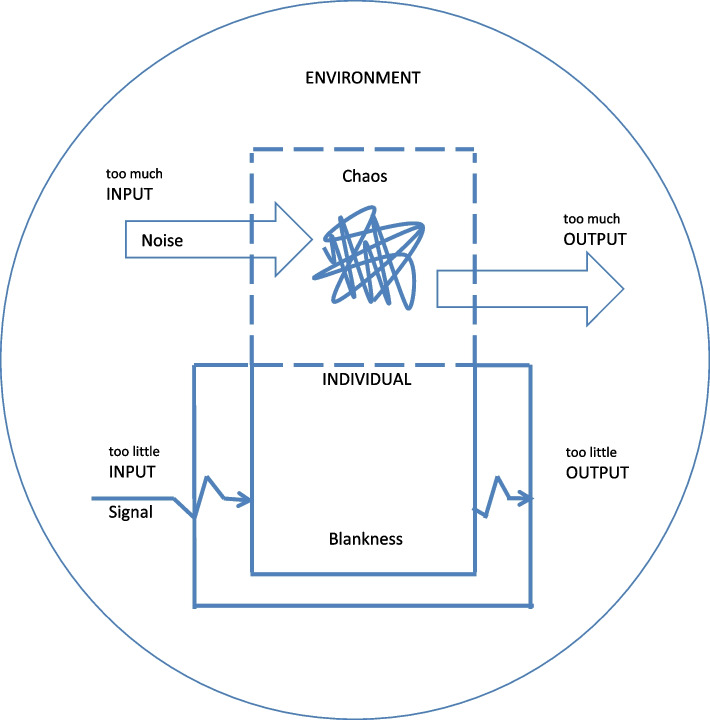
I find it hard to regulate what I take in and what comes out

Two different sub-themes emerge from the interviews revolving around how the adolescents assimilate and process information, what is happening in their minds, and their ability to initiate and regulate their actions. To capture the two sub-themes a model was created (see Fig. 1) in which the sensory impressions that individuals take in are referred to as input, and their actions as output. In some cases both of these two ways of working were recounted by the same individuals. The difficulties in regulating level of input and output can result in the experience of being either off or on.

#### Inner chaos

In the sub-theme inner chaos adolescents portray what is happening in their minds both as a jumble of impressions, and as thoughts rushing around. Another aspect of the internal turmoil is dealing with intense emotional reactions.

*“So it becomes… just that not being able to sort things in the right order*,* it very easily causes chaos…It simply becomes chaos in the brain.” #16*.

In inner chaos input is characterised by an overloading amount of impressions, with a limited ability to filter these impressions. This kind of constant “noise” makes it difficult to distinguish the signals you want to pay attention to.

*“Yes*,* that’s right*,* it’s difficult to control your… what’s it called… concentration like*,* now I’m going to focus on this*,* but at the same time*,* you take in so much else as well*,* like*,* you like… if you say that someone who doesn’t have ADHD can kind of filter so they’re just: okay*,* now I concentrate on this*,* like*,* what’s on the board*,* and what the teacher says*,* while someone with ADHD can… doesn’t have this filter like so they kind of see everything…”* #13.

In inner chaos output refers to behaviours that the adolescents themselves view as ill considered and “too much”. Unintentionally, actions “slip out” through the holes of the permeable filter between themselves and the environment.

*“Well*,* you say something stupid that you don’t even mean*,* and you don’t even know that you’ve said*,* and then people can get upset.”* #20.

#### Blankness

In the sub-theme inner blankness the adolescents talk about how their mind is going blank, accompanied by an inability to make content stick, or to access content that they know should be there:

*“If the teacher asks a question and someone in the class is supposed to raise their hand and answer then… and I may know what it is*,* but I can’t… I have it on the tip of my tongue but it never comes out because I do not know how to say it. ”* #14.

Descriptions about input refer to how the signal never gets through the filter. There is a difficulty of directing attention, taking in the right signal and being able to process information as desired.

*“… to have to do something according to instructions that I heard kind of a minute ago it’s so super stressful*,* because I am… it feels like I kind of… I can not focus sometimes because it’s like this “bah*,* shit*,* I just can’t “…"*#12.

It is like the inner intent gets stuck on the way, with difficulties initiating and completing actions, leading to “to little” output. This difficulty appears to sometimes be related to an inability to prioritize, sometimes to the lack of energy and sometimes to an inner resistance.

*”The thing that affects me most in everyday life is that I have difficulty getting things started. That’s probably the biggest problem that I think is bad.”* #4.

#### I am either off or on

Related to the difficulties regulating the level of input and output is a description of being either off or on. There is a shortage of flexible customized variability regarding the intensity of different internal and external responses. Instead, both overreactions and underreactions are common, with regards to regulation of attention, emotions, energy levels or stress.

*“My difficulties*,* it’s probably those typical ADHD things*,* that my mood is up and down all the time*,* there is no middle ground*,* not at all. It’s always from one to one hundred*,* all the time and it’s either or. It is probably my biggest problem - that I can’t handle that.” #8*.

A characteristic that some experience as positive and that they think distinguishes them from others is a sudden ability to concentrate very well in certain situations at times when they find something interesting or enjoyable. When this hyperfocus is on, almost nothing can disturb them. It can be conceptualised as a sudden possibility to close the overly permeable filter, shut out the “impression noise” and deliberately canalise the energy. Hyperfocus stands in stark contrast to the first two sub-themes presented, for once something is functioning effortlessly.

*“Just this*,* not being able to really focus on one and the same thing for long periods of time*,* or not having interest in anything*,* and just simply not listening. And then*,* when you finally find something that you like*,* that becomes… then you focus on that one hundred per cent.”* #15.

On the other hand, hyperfocus can turn from something positive to something negative, as it sometimes leads to being completely shut off from the outside world and even forgetting to both eat and sleep. The filter is closed and therefore it becomes difficult to pay attention to new signals and switch attention to them.

### I’ve really struggled but it does not seem to work

A central theme across interviews is an inherent exertion and mental strain linked to living with ADHD. The adolescents struggle to get their everyday life together, both at school and at home. Their undertakings are often delineated as both energy-intensive and fruitless.

#### My way of being works against me

The described way of functioning, with difficulties in regulating information intake, thought processes and behavior, is perceived by the adolescents as stressful, strenuous, and impairing.

*“I don’t care*,* I do not have the energy. Like that*,* because I kind of have had no stamina. I’ve really struggled*,* but it does not seem to work.”* #18.

A lot of things require more effort to carry out because of an inner resistance containing an emotional aversion, a mental state opposite to motivation. Some interviews involve a description of doing almost anything to avoid or postpone doing something aversive, like homework, and the adolescents evaluate themselves negatively in relation to others.

*“No*,* I usually*,* I kind of decided to do things with structure beforehand. I’ve sort of decided that I can’t sit down at the computer or the TV until I have done it. But then instead I just sit and do nothing. Or subconsciously*,* for example*,* when I have schoolwork to do*,* I clean my room instead*,* which I would never do otherwise. It’s kind of like*,* I do anything else.”* #4.

Another aspect described as demanding by some, adding to the experience of increased effort and loss of control, is a difficulty in being flexible. Being able to carry out things that are unforeseen, unplanned, and not part of the usual routines is described as challenging.

*”When things goes beyond my planning or something like that. Then I’ll become super stressed and destroy everything around me*,* relationships or mirrors - just everything.”* #8.

Several of the adolescents find it difficult to describe what it is like living with ADHD. The lived experience is difficult to capture and put into words, because they know of nothing else. They find it hard to distinguish traits of ADHD from who they perceive themselves to be.

”*I don’t know*,* it’s hard to say. But precisely this*,* that I kind of… altogether… it feels like it is a part of me. Who I am.”* #4.

#### I get stressed out, anxious and exhausted

The adolescents frequently mention stress and many had a hard time differentiate the experience of stress from symptoms of ADHD. As a result of the stress and mental strain in everyday life the adolescents express that they become anxious or drained of energy.

*“I do not feel well*,* because I get so tired of just being*,* so will I just have to rest now for one day.”* #7.

Anxiety is often mentioned together with stress, sometimes as either one triggering the other, sometimes as two facets of the same experience.

*”Usually when I get very stressed*,* I get a lot of anxiety*,* which means I can’t relax and I can’t breathe”#*15.

Going to school for a whole day is described by most as very strenuous and several young people also say that they miss a part of life, as the energy is already consumed after the school day. There is also a desire to be as perseverant as other young people.

*“No*,* I really did not understand why … how the others could do everything. They were superheroes in my eyes. How can they like go to school*,* exercise*,* kind of like play football and then meet friends on top off that. I went to school*,* then I kind of caved in when I got home.”* #16.

### ADHD impacts my vitality and my relationships

Different consequences of ADHD are described by the adolescents. Inner stress and exhaustion has already been mentioned. In the material there are also repeated descriptions of a relational consequences where the adolescents are disturbing to and disturbed by others. With the high strain and repeated failures come self-accusations and a tendency of giving up.

#### I am tiresome to others, others are tiresome to me

Interpersonal consequences of ADHD are disclosed in several of the interviews. The interviews recount how ADHD symptoms and related behaviour can be perceived as tiresome to the environment, causing others to be irritated. Interrupting others, talking excessively and making comments that others are offended by, are some of the examples mentioned. This involves an experience of being a nuisance to teachers and other adults because of too much output.

*“I have always*,* throughout my schooling*,* had problems with teachers and so on. Because teachers don’t like me and that is natural*,* it’s natural that a teacher flips out on a student who sits and talks straight out every lesson*,* and who runs around like a headless chicken”.* #2.

Another phenomenon is that others may be disturbed or displeased because of a blankness or lack of output, for instance when the adolescents are not doing what is expected of them. Others can interpret this behaviour as negligence. Here the adolescents bring up examples of not doing their part in group assignments, not replying to text messages, and not listening when spoken to. The inability to direct attention and retrieve information can also lead to misunderstandings, a failure to take note of social signals and the risk of being perceived as aloof. Furthermore, deficiencies in time perception, manifested as not showing up in time, can cause annoyance.

*“… I rarely have the energy to write with people*,* for when I have the time to do it*,* then I’m too tired. … so there are some people that I haven’t spoken to in like a month*,* who normally spend time with. And they just go “but why haven’t you answered” and I just say “ehh*,* sorry”.* #18.

At the same time the adolescents describe how other people can be tiresome to them. They describe an easily triggered irritability and anger and a tendency of overreacting to small disruptions or misunderstandings. This can in itself comprise a strain on relationships.

*”Since I’m more easily affected by general impressions*,* my friends tend to get… well*,* they usually get a taste of my irritation and my anger. So I always apologize to them and say that it’s not them I’m angry with.”* #15.

As both life in general and social interactions can be more tiring for these adolescents, they sometimes do not find the energy to socialize with peers. This in turn can lead friends to misinterpret their intentions, lose patience, and withdraw.

#### I resign and blame myself

A sense of failure and resignation emerges in the stories together with self-blame. Feelings of helpnessness, a lack of control and sometimes even devastation. Some describe easily getting feelings of guilt after accidently doing something wrong. Others recount how stressful circumstancies can be so overwhelming it leads them to give up and do nothing.

”*When I become so stressed that I can’t do anything*,* then I lie down and stop caring and I feel like I’m a failure.”* #7.

Helplessness and perceived failure leads to feelings of entrapment and resignation. Taken together this downward spiral can induce low mood.

*”…So I get more and more depressed*,* because I feel that “now I can’t manage to do anything”.* #19.

### I can be successful and things are getting better

ADHD is not only described as a challenge, but also as an asset. It is seen as a source of creativity, vitality and motivation. And the challenges it poses can be overcome.

#### When I am vigorous and creative

A special kind of energy, motivation or drive is recurrently expressed by the adolescents as something that both makes them and their lives more interesting. We gain insight into an elate state of mind.

“*Well there are many positive things. There are not as many advantages as there are disadvantages*,* but for instance that you feel more emotions is a… for me it can be a drive*,* that like… you… you feel like a… if you feel motivated or happy then like… then it’s really like there’s a fire burning inside you in some way.”* #12.

Several of the participants describe that they often feel extremes in emotional experiences, both positive and negative. Some find it positive to have strong emotions, because they give life extra spice.

*“When I really do something or feel something*,* I really do it with all of my heart. So*,* I’m not half in love*,* for example*,* or I’m not half happy*,* it is not like that. No*,* then I really feel very well or am very much in love with someone.”* #8.

A related positive aspect of ADHD seems be creativity as in having many ideas and arriving at unconventional conclusions.

*“Yes*,* I am very creative. And so I get new ideas all the time. Although half of them are completely idiotic*,* I constantly get a lot of good ideas. So I have a lot of energy and when I put it into things I should do or will do*,* it works out great.”* #5.

#### When I use the inner stress or strong emotions as motivation

When the adolescents talk about hyperfocus or a special kind of energy it is conveyed with positive connotations. In some instances, they describe approaches they use to put themselves in this state. A generic strategy of using emotions as a driving force to accomplish something emerges. They portray an interplay between motivation and time pressure, where on-going internal stress can be transformed into motivation and the production of output if time for a task becomes scarce. Procrastination becomes a strategy, to create sufficient motivation and a good result is attributed to the internal pressure they managed to create.

*“I mean I do care*,* if I had a choice I would do it*,* finish it. But the thing is*,* if I had finished it in time*,* it probably wouldn’t have turned out as good. So I’d rather hand it in late and do it well*,* than hand something in on time that I’m not completely happy with. Because the stress helps me to do better. Since then I feel that “now I really have to do this”. When it’s almost a life-or-death situation*,* then I do it.”* #5.

Also, determination to succeed can have evolved from emotions stemming from adversity and hardship.

*”And there are things like when I was bullied*,* when I was younger*,* so instead of just saying “okay*,* now it’s like this” and losing hope in everything*,* I used my anger that I had towards them and like transformed it into this kind of motivation*,* that like this “I’ll be better than the kids at my school”*,* “I’ll… be better” like that”* #12.

#### With time for recovery, good strategies and the help of medicine

Many view the home as a haven for recovery where they are met with lower demands, an understanding and a willingness to help and solve problems.

*”After all*,* I have my room and my bed where I spend almost all of my time. It’s like my safe place. So yes… then I have my parents who… especially my mother*,* she realizes quite quickly when something is wrong*,* so she can usually help quite a lot.”* #15.

The need for recovery and breaks is also highlighted by some in other situations such as during the school day and during free time.

Some of the adolescents talk about strategies they have evolved to have things work better. To ruminate less, to go ahead and do things irrespective of what you think or feel are strategies mentioned. Also, to be aware of your way of functioning and of what is really important to you.

*”… like you have to be aware of your values*,* how to do things*,* what you kind of want to achieve*,* because otherwise you will let your impulses rule*,* and then nothing will work out*,* kind of.”* #12.

A recurring topic in the interviews is how medical treatment facilitates life and everyday functioning.

*“I notice a big contrast between what I am like with medicine and without medicine*,* it really is… my medicine it’s like this… everything works*,* it really works"#*12.

Occasionally negative effects of the medication are mentioned such as increased irritability and change of weight.

#### Certain symptoms have decreased over time

Furthermore, an experience is noted that certain typical ADHD symptoms have decreased or changed over the years. For example, hyperactivity may have decreased.

*“It was already noticeable when I was younger*,* with the concentration and sometimes I had great difficulty sitting still*,* but it was mostly when I was younger. Now it is mostly just the concentration that is the problem. (…)”* #14.

Some have noted that emotion regulation skills and impulse control have improved over time.

*”I got it when I was five or six so I’ve had it for quite a while now. It was probably more visible on me when I was younger. I was very moody*,* edgy*,* felt*,* well it was more that people commented a lot and I guess I didn’t feel too good about that.”* #9.

## Discussion

This qualitative study aimed to explore adolescents’ perceptions of how it is to live with ADHD. The analysis resulted in four themes: [1] I find it hard to regulate what I take in and what comes out [2] I’ve really struggled but it does not seem to work [3] ADHD impacts my vitality and my relationships, and [4] I can be successful and things are getting better. The first three themes are closely connected to one another. Theme one covers details of how the problems with self-regulation were perceived to be interlinked by the adolescents with ADHD, theme two the primary consequences of these self-regulatory problems such as stress, anxiety and loss of control, and theme three delineates wider secondary consequences such as exhaustion, depressed mood, challenges in social interaction, self-accusations and a tendency of giving up.

In theme one a preliminary model is sketched of how the difficulties with self-regulation are perceived by the adolescents. The model encompasses cognitive, emotional and behavioural aspects. The value of the model is not primarily theoretical or causal, but serves as an illustration of the adolescents lived experiences. It should be noted that the two different modes - inner chaos or blankness – may be closely linked where both an inner chaos of thoughts and emotions and an inner blankness could result in too little output. The sub-theme ‘I’m either off or on’ touches upon ADHD as a double-edged sword, resulting not only in costs, but also in benefits, an aspect found also in previous studies (e.g. [28]). More specifically the ‘being on’ position has positive sides to it, elaborated further in theme four under the headings ‘When I am vigorous and creative’ and ‘When I use the inner stress or strong emotions as motivation’. The description of hyperfocus – when the attentiveness, motivation, level of energy and capacity for initiative are optimized – show similarities to the concept ‘Flow’ [48]. ‘Flow’ has been defined as an intrinsically rewarding subjective state of ”intense experiential involvement in moment-to-moment activity” [48, page 230].

In terms of primary and secondary consequences of living with ADHD, a striking aspect of the findings in this study is how profound the emotional impact was described to be. Stress and loss of control emerged as central aspects of living with ADHD, and, by extension, becoming anxious, exhausted, and depressed – conditions that have been associated with ADHD [11]. Another emotional aspect frequently mentioned is easily evoked irritability, which can lead to complications in social situations. The emotional reactivity is however two-sided and also contains an element of elevated mood, which was appreciated by the participants. Indeed, children and adolescents with ADHD have increased vulnerability for emotional problems such as irritability, anger or stress [17]. Research suggests that up to 50% of children and adolescents with ADHD present with a co-occurring externalising condition, such as oppositional defiant disorder or conduct disorder, and up to 40% have internalising conditions such as depression or an anxiety syndrome [49]. Across the lifespan, the type of co-occurring conditions may change, with affective diagnoses becoming more prevalent in adolescence and adulthood [11].

Our findings are in line with the growing literature on emotional dysregulation as an important aspect or symptom dimension of ADHD with increased hyperactivity/restlessness, temper, affective lability, and emotional over-reactivity [50]. In the sub-theme of inner chaos, intense emotional reactions, such as anger, was often described as an element leading to too much (impulsive) output. Similar results were mentioned in Clancy et al. [35] p.234) were “a turbulent inner atmosphere of difficult emotion” was causing the adolescents to being unable to focus on anything else, and to seek distraction. These finding can be hypothesis generating with emotional regulation being one of several possible mechanism underlying the core diagnostic symptoms of ADHD. Beyond the core symptoms of ADHD, emotional dysregulation has been found to be associated with additional functional impairment [9], as well as with co-occurring problems of both an externalising and an internalising type [51]. Consequences related to the features outlined in theme one included statements that too much output (impulsive behaviour or hyperactivity), as well as to little output (e.g. not replying to messages), can be perceived as tiresome by others. It is known that social interaction can pose a challenge for young people with ADHD [5] and our findings underpin the process of how the interaction becomes a problem. This is information of value both for differentiating social impairment in ADHD from that found in autism, and when presumably outlining social skills training specific for ADHD.

The fourth theme deals with empowerment, strategies for obtaining control and depicts a timeline with symptoms declining over time. Control or the lack of control is however a feature that recurs throughout the results, and failed attempts of regulating the level of energy, motivation, emotions and behaviour all point in the same direction. A salient experience is how the adolescents’ give everything they have and still cannot make life work. Previous studies on young people’s experience of having ADHD have also touched on aspects of loss of control. Krueger and Kendall [35] describe that this was handled differently by girls and boys in their material where the boys left the triggering situation over which they had no control or used aggression as a means of power, while the girls experienced failure and turned it against themselves in a manner which made them passive and resigned [35]. In Clancy et al. [34] participants described an ‘all or nothing’ approach to the search for control in their lives. Most of the participants shared a dread of uncertainty and a need to ‘know’ things for sure in order to feel safe. There were also recurrent descriptions of the repulsion of being controlled by others, an experience shared with the boy participants in Krueger and Kendall [35] who wanted to be ’left alone’ and believed that others were attempting to take over their power.

The other end of the spectrum is empowerment. Interestingly, the adolescents describe certain aspects of their ADHD-specific way of functioning as empowering in itself. They perceive that they have a gift of vigour and creativity that makes them stand out and that they sometimes can use this power deliberately, as outlined in sub-theme ‘When I am vigorous and creative’ and ‘When I use the inner stress or strong emotions as motivation’. In the findings different means of obtaining control are described - medical treatment, coping strategies and support from the environment. Cooper and Shea [30] found that there were two very different ways of looking at the link between medical treatment and control among adolescents with ADHD. A view of medicine as a means of gaining control and a competing view of medicine as a means by which one was controlled by others. Earlier studies have found that adolescents described who they were in reference to their ADHD symptoms, instead of holding an identity distinct from the disorder (e.g. [35, 52]. This tendency is also found in our material in the sub-theme ‘My way of being works against me’. Given that adolescents with ADHD can have difficulties in differentiating traits of ADHD from their perceived identity, e.g. that ADHD is what makes them be the unique fun person they are, these circumstances can perhaps bring some further understanding to why many adolescents stop taking their medication. This perception that the medication affected the sense of self was also discussed by Cooper and Shea [31] as the ‘real self’ in their interviews was delineated as the non-medicated self. This factor may be important to acknowledge when discussing initiation of ADHD medications among this population.

### Methodological considerations

There are some methodological aspects of this study that deserve consideration. The sample consisted to a large part of participants with a combined presentation subtype of ADHD, with an on-going drug treatment. Thus the predominantly inattentive presentation subtype was not well represented, as is often the case in clinical samples in comparison to the population [15]. Further, the resistance towards medication known to occur in adolescence was less likely to be captured in the sample recruited than in the population. The sample was recruited from a group therapy based on Dialectical Behaviour Therapy [40] a fact that may have caused some selection bias, given that not all adolescents with ADHD would be interested in partaking in such a treatment. It can also be expected that the treatment had some influence of the adolescents’ well-being and view on their situation. This fact is double-sided as it can also have made them more aware of themselves and their experiences. In terms of gender there was a slight overrepresentation of girls in the sample, although ADHD is twice as common in boys. In studies evaluating psychological treatment of ADHD, girls however tend to be overrepresented [53, 54]. Considering the overrepresentation of girls we also conducted a sub-group analysis. The sub-group analysis showed that both boys and girls mentioned all themes and sub-themes.

There are limitations to the study approach in that data saturation was not used in the recruitment procedure, and that member checking was not used. One reason for not including member checking was the amount of time that passed from the interviews to the transcriptions and analysis. Regarding sample size, all the sub-themes and aspects brought up in the last two interviews had already been covered in the previous interviews giving some indication of data saturation. The researchers of this study were health care professionals with extensive experience in the field of child and adolescent psychiatry. This fact connects to their credibility, but also to their specific pre-understanding of the phenomena. Measures were taken to strengthen the trustworthiness of this study. A thick detailed description has been provided with regards to the site, participants and methods used for data collection and analysis. With regards to confirmability examples of the process of going from codes to sub-themes and themes has been provided and with regards to referential adequacy the result section contains a high number of illustrating quotes.

## Conclusion

Adolescents with ADHD experience problems with self-regulation with regards to cognitive, emotional and behavioural processes. They describe being either on or off. This difficulty to self-regulate is related to primary and secondary consequences such as stress, anxiety, loss of control, and by extension exhaustion, depressed mood, challenges in social interactions, self-accusations and a tendency of giving up. The experience of ‘being on’ has positive connotations of vigour and creativity. The challenges related to ADHD can to some extent be overcome with time for recovery, coping strategies, and medical treatment. Symptoms of ADHD can also decrease over time.

## Supplementary Information

Supplementary Material 1.

## Data Availability

The data generated during the current study is not publicly available due to restrictions in Swedish legislation, but may be available from the corresponding author on reasonable request.

## Abbreviations

ADHD: Attention deficit/hyperactivity disorder
ASRS: A The Adult ADHD Self-Report Scale for Adolescents
COREQ: The consolidated criteria for reporting qualitative studies
MINI Kid: The Mini International Neuropsychiatric Interview for children and adolescents
