# Supplementary material for: I’ve really struggled but it does not seem to work: adolescents’ experiences of living with ADHD – a thematic analysis
Source: BMC Psychol. 2025 Jan 27;13:75. doi: 10.1186/s40359-025-02350-7 (PMC11773757; doi:10.1186/s40359-025-02350-7)
Supplement: Supplementary file 1 — Supplementary Material 1. [file 40359_2025_2350_MOESM1_ESM.docx]

**Supplement material.** Interview schedule

**ADHD**

*What is it like, having ADHD?*

*What is good or bad with ADHD?*

Use open-ended follow-up questions to let participants elaborate on their answers

Explore received answers in relation to home, school, relationships and wellbeing

**Stress**

*How do you experience and perceive stress?*

Use open-ended follow-up questions to let participants elaborate on their answers

Explore received answers in relation to home, school, relationships and wellbeing.

**Treatment**

*How did you experience participating in a group?*

*What did you appreciate with the treatment?*

*What did you dislike with the treatment?*

*Did the treatment result in any changes?*

*What aspects of the treatment will you benefit from?*

*Should anything in the treatment be different?*

Use visual support (e.g., themes of the treatment) to help the participants remember the content.

Use follow-up questions when appropriate
